# Electrochemical Investigations of *Galium verum* Ethanolic Extract as a Steel Corrosion Eco-Inhibitor in the Acid Media: An Unexpected Versatility of Plant Chemistry

**DOI:** 10.3390/ma18092078

**Published:** 2025-05-01

**Authors:** Anca Cojocaru, Gabriela Elena Badea, Ioana Maior, Simona Dzitac, Oana Delia Stănășel, Mioara Sebeșan, Camelia Daniela Ionaș, Petru Creț

**Affiliations:** 1 Department of Inorganic Chemistry, Physical Chemistry and Electrochemistry, Faculty of Chemical Engineering and Biotechnology, National University of Science and Technology Politehnica Bucharest, 313 Splaiul Independentei, 060042 Bucharest, Romania; anca.cojocaru@chimie.upb.ro; 2 Department of Chemistry, Faculty of Informatics and Sciences, University of Oradea, 1 Universitatii Str., 410087 Oradea, Romania; 3 Department of Energy Engineering, Faculty of Energy Engineering and Industrial Management, University of Oradea, 1 Universitatii Str., 410087 Oradea, Romania; 4 CNCG-National Centre of Geothermal Research, University of Oradea, 1 Universitatii Str., 410087 Oradea, Romania

**Keywords:** *Galium verum* ethanolic extract, polyphenols, chromatography, steel corrosion eco-inhibitor, chlorohydric acid, statistics

## Abstract

Corrosion inhibitors are substances that reduce or eliminate the corrosion of a metal in a certain environment. Corrosion inhibitors act by several mechanisms, including adsorption, film formation, passivation, and oxygen scavenging. Due to their toxicity, classic corrosion inhibitors affect the environment. Therefore, in recent years, more and more studies have focused on the development of eco-friendly inhibitors for the environment. In this study, ethanolic extract of *Galium verum* (GV) was tested for the inhibition of steel corrosion in 1 M HCl medium using electrochemical methods: open circuit potential (OCP), potentiodynamic polarization (PP), and electrochemical impedance spectroscopy (EIS). Reverse-phase liquid chromatography (HPLC) and gas chromatography mass spectrometry (MS-GC) previous studies state that GV extract contains polyphenols and other chemical species responsible for the inhibitory effect. Corrosion investigations have highlighted the influence of the concentration of the GV extract, in the range of 50 ÷ 400 ppm G.A.E./mL, as well as the influence of temperature in the range of 20 ÷ 50 °C. The corrosion inhibitory efficiency of the *Galium verum* ethanolic extract had a maximum value of 91.82% for a concentration of 400 ppm polyphenol content, demonstrating the inhibitory potential of this green product in an acidic environment for mild steel. Statistical calculus on the obtained values of EIS inhibitor efficiency showed that the effect of the extract becomes stronger at higher concentrations.

## 1. Introduction

The natural process of corrosion happens when metals or alloys interact with their surrounding environment. It can cause damage to metallic structures, leading to expensive maintenance, repair, and replacement costs.

Metals and alloys can be protected using a variety of techniques (for example, steel, copper) against corrosion depending on different aspects, such as metal, medium, temperature, costs, and others. One way to minimize the effects of corrosion is through the use of corrosion inhibitors. They cause the rate of corrosion to slow down.

Corrosion inhibitors are different; they can be organic or inorganic inhibitors. Organic inhibitors contain heteroatoms (N, O, or S) that can adsorb onto metal surfaces. Inorganic inhibitors are compounds containing anions, cations, or oxides that form a protective layer on the metal surface. Both types of inhibitors can be used in different environments, depending on the type of metal and the corrosive agents present.

The way adsorption inhibitors work is by creating a barrier, a film that stops further corrosion. By creating a thin protective film on the metal surface, film-forming inhibitors prevent the metal from coming into contact with corrosive agents. By creating a passive layer of oxides on the metal surface, passivation inhibitors stop additional environmental reactions. By lowering the quantity of oxygen accessible for the corrosion reaction, oxygen-scavenging inhibitors lower the rate of corrosion.

Corrosion inhibitors can be used in a variety of applications, including oil and gas production, metalworking fluids, cooling water systems, and construction. In oil and gas production, corrosion inhibitors are used to protect the steel pipes and equipment from corrosive gases, for example, CO_2_ or H_2_S. In metalworking fluids, corrosion inhibitors can prevent the corrosion of metal parts during machining and increase the fluid’s lifespan. In cooling water systems, corrosion inhibitors can protect the metal pipes and equipment from corrosion caused by microorganisms and mineral deposits. In construction, corrosion inhibitors can protect steel reinforcing bars from corrosion caused by exposure to chloride ions.

Corrosion inhibitors are essential in protecting metallic materials from the effects of corrosion. The efficiency of a corrosion inhibitor is determined by the type of inhibitor employed, its concentration, the kind of metal, and the presence of corrosive chemicals. Different types of corrosion inhibitors may be combined for better protection. Corrosion inhibitors have several applications in various sectors and play a significant part in preventing costly corrosion damage.

Due to the toxicity of the substances used in the past as inhibitors (such as chromates, nitrites, and phosphates) and the increased awareness of the dangers to the environment, there is an increasing scientific interest in finding new inhibitors that are both efficient and environmentally friendly. Green inhibitors based on extracts made from different parts of a plant open a great perspective for the development of an environmentally friendly industry of corrosion inhibitors. Due to the great variety of plants, in the past years many reviews and original studies were published regarding corrosion inhibitors for steel protection in various media [1,2,3,4,5]. Some of the most recent and relevant research for mild steel in an HCl environment includes *Ammi visnaga* L. extract [6], *Xanthium strumarium* leaves extract combined with iodide ions [7], *Syzygium cumini* leaf extract [8], *Cinnamoum tamala* leaves extract [9], *Piper nigrum* (Black pepper) [10], procyanidin B2 compound obtained from *Uncaria laevigata* [11], *Crataegus oxyacantha* leaves extract [12], *Ceratonia siliqua* L. seeds extract [13], *Ocimum basilicum* seed extract [14], winged bean extracts [15], *Cannabis sativa* L. seed oil [16], *Eucalyptus camaldulensis* leaves [17], *Chamomile flower* extract [18], *Akebia trifoliate koiaz* peels extract [19], walnut fruit green husk extract [20], *Paederia foetida* leaves extract [21], *Thaumatococcus daniellii* [22], *Sonneratia caseolaris* leaf extract [23], and *Sargassum algae* [24]. These are only a few of the recent publications in international journals regarding the use of natural products for protecting metals against corrosion. Different phytocompounds were the cause of the corrosion inhibition effect [2,5,25,26]. Polyphenols are known to have high biochemical antioxidant effects [27,28], which make them potential green inhibitors by two possible mechanisms: being an actor for oxidation reaction and/or adsorbing on the metal surfaces, thus becoming a corrosion inhibitor.

After an extensive review of the literature, including research on databases, there were not found any studies exploring the use of *Galium verum* as a corrosion inhibitor.

Recent studies show that *Galium verum*, from spontaneous flora [29,30,31], has a rich content of bioactive compounds such as phenolic compounds, flavonoids, iridoid glycosides, essential oils, and antioxidants [32,33,34]. The plant’s phytochemicals contribute to its multiple bioactivities and potential applications [35]. Different extraction methods have been investigated, such as reflux extraction [36], maceration [37], ultrasound-assisted extraction [38], and microwave extraction [39]. Pharmaceutical and cosmetic applications, such as immunomodulatory effects [40], antioxidant and anti-inflammatory properties [38,40,41,42,43], cytotoxic effects [39,41,44], healing and antimicrobial effects [43,44], or dermatocosmetics [38], have been investigated. The majority of these activities are linked to flavonoids and polyphenols [45,46].

Electrochemical methods most used to investigate corrosion inhibitors are gravimetry [28,47,48], potentiodynamic polarization curves, and electrochemical impedance spectroscopy [48,49]. Moreover, the electrochemical approaches of green inhibitors for metals in acidic environments, especially in HCl, methods for analyzing the corrosion inhibitory effect of natural plant extracts included computational investigations [50], quantum mechanics [51], surface analysis [47,49,52], FTIR (Fourier transform infrared spectroscopy) [49,52,53], or in-depth analysis of experimental and theoretical approaches [54,55,56,57,58]. Some authors also explained the high efficiency of polyphenol plant extracts due to the presence of heteroatoms and π-electrons [53].

Corrosion in acidic environments is common in industrial applications, and HCl is frequently used in chemical cleaning and pickling, making it a relevant acid for the tests. Using a 1 M HCl solution as a standard for acid corrosion studies on mild carbon steel is justified for several reasons, such as reproducibility and industrial relevance.

The aim of this original study is to propose a new type of green inhibitor, *Galium verum* (GV) ethanolic extract, for carbon steel in 1 M HCl solution. Electrochemical methods (open circuit potential—OCP, potentiodynamic polarization—PP and electrochemical impedance spectroscopy—EIS) were used for investigation. GV extract was not tested before as a corrosion inhibitor. In a previous study [28], a chemical characterization of *Galium verum* extracts using analytical techniques was completed. Reverse-phase high-performance liquid chromatography (HPLC) identified and measured the presence of polyphenols: gallic acid, catechin, vanillic acid, caffeic acid, kaempferol, quercetin, and umbelliferone. Gas chromatography-mass spectrometry (GC-MS) studies highlight compounds including alcohols, phenols, fatty acids, hydrocarbons, esters, and other aromatic compounds. Among the substances usually identified in plant extract to be corrosion inhibitors, polyphenols are considered the main actors in limiting the corrosion rate of metals.

While *Galium verum* has been studied in other contexts, predominantly for its phytotherapeutic properties, the application as a corrosion inhibitor is novel and has not been previously reported.

The primary objectives of this research are to analyze the efficiency of GV ethanolic extract on steel corrosion rate in an acidic environment and to determine the optimal temperature and concentration for the most effective corrosion inhibition effect using rapid electrochemical techniques.

## 2. Materials and Methods

In order to investigate the effectiveness of different natural plant extracts as corrosion inhibitors on the corrosion rate of steel in an acidic environment and to determine the optimal concentration of the GV extract as a corrosion inhibitor, electrochemical methods (open circuit potential—OCP, potentiodynamic polarization—PP, and electrochemical impedance spectroscopy—EIS) were used for investigation.

### 2.1. Materials

The dehydrated botanical specimen, purchased from the market, was used to prepare GV extract. Chopped dried aerial parts of the plant were used for phytochemical extraction using Soxhlet equipment. Ethanol (Merck KGaA, Darmstadt, Germany) with analytical grade was used for reflux extraction of GV. An amount of 35.81 g of the dried plant was used in 260 mL of ethyl alcohol in 3 h reflux extraction. The extract of GV was stored at 4 °C before use. The concentration of reflux GV extract solution was 2000 ppm gallic acid equivalent (G.A.E.)/mL, corresponding to the total polyphenolic content of the extract, determined with the Folin–Ciocalteu method, also used in previous related studies [27,28].

### 2.2. Chromatography Methods

#### 2.2.1. RP-HPLC Chromatography

An HPLC ACME 3000 Young Lin Instrument (Young Lin Instrument Co., Ltd., Anyang, Gyeonggi-do, South Korea) with an SP 930D module and a UV 730D detector module was used to determine phenolic compounds.

A mobile phase consisting of methanol, water, and acetic acid at a ratio of 300:700:2 was used to carry out the chromatographic separation under isocratic conditions. One milliliter per minute was the elution flow rate. For the separation, a 150 mm long, 4.6 mm internal diameter reverse-phase YMC-Pack ODS AQ column (YMC Co., Ltd., Kyoto, Japan) was employed. At room temperature, 0.2 µL of each sample was put into the chromatograph.

#### 2.2.2. GC-MS Chromatography

A Thermo GC-MS system (Model Trace 1310 ISQ7000) (Thermo Fisher Scientific, Waltham, Massachusetts, USA) was used to perform the GC-MS analysis of the Soxhlet extract that was extracted from the aerial parts of *Galium verum*. An HP-5MS capillary column (Agilent Technologies, Santa Clara, California, USA), which was 30 m long, 0.32 mm in internal diameter, and coated with a 0.25 μm film thickness, was connected to this apparatus.

An electron ionization device with an ionization energy of 70 eV was used for GC-MS spectroscopic detection. The carrier gas, helium, flowed at a velocity of 30 cm/s. A 1 μL injection volume was chosen.

The injector and mass transfer line temperatures were kept at 290 °C and 220 °C, respectively. Initially, the oven was set to 45 °C for one minute. After that, it was raised to 250 °C at a rate of 5 °C per minute. The temperature was kept at 250 °C for 5 min. In split mode, samples were injected with a split ratio of 120:1 after being diluted to 1 μL.

### 2.3. Electrochemical Methods

Inhibition efficiency was investigated in solutions obtained by adding to a volume of 50 mL of 1 M HCl of different volumes of GV extract, corresponding to amounts of polyphenols of 50, 100, 200, 300, and 400 ppm G.A.E. GV extract was added into the corrosion cell with the help of an LLG automatic microliter pipette. The concentration of 1 M HCl is considered to remain constant because the added volumes of inhibitor were more than 100 times smaller.

A commercially available mild carbon steel, S235 type, was used for testing. The elemental composition of S235 C-steel alloy is as follows: C: 0.22, Si: 0.05, Mn: 0.6, Ni: 0.3, S: 0.04, P: 0.04, Cr: 0.3, N: 0.012, Cu: 0.3 (weight %) and the remainder is Fe.

The testing electrodes were steel plates with 1 cm^2^ exposed surface area. The exposed surface was polished with different sizes of sandpaper (P600, P2000, and P2500) to obtain a smoothed surface and then washed with distilled water and acetone.

The electrochemical measurements were performed using a Voltalab 40 potentiostat/galvanostat (Radiometer Analytical, Villeurbanne, France), coupled with VoltaMaster 4.0 software for data acquisition and processing. The used electrochemical system was a three-electrode arrangement with carbon steel S235 (1 cm^2^) as the working electrode, platinum gauze as the counter electrode (5 cm^2^ active area), and Ag/AgCl saturated electrode (SSCE) as the reference in a thermostated electrochemical cell for temperature control.

Before each measurement, the working electrode was polished with emery paper of increasing grade (600–2500), washed with distilled water, and dried. Before each electrochemical measurement, the open circuit potential (OCP) of the working electrode was recorded for 30 min in acidic medium with and without additions of *Galium verum* extract in order to reach the equilibrium state. The Tafel potentiodynamic polarization (PP) measurements were obtained with a sweep rate of 20 mV/min, starting from cathodic potential towards the anodic region. For every electrochemical experiment, a minimum of 3 trials were performed. The best reproducible result of a trial set is selected, for each concentration and temperature.

The inhibition efficiency (*IE*) was determined from Tafel plots using the Formula (1):

(1) $$IE=\left(1-\frac{i_{corr}}{i_{corr}^{0}}\right)\times 100\;\%$$

where *i*^0^*_corr_* and *i_corr_* (µA/cm^2^) are corrosion current density values in 1 M HCl solution, without and with GV extract.

The electrochemical impedance spectroscopy (EIS) measurements were conducted (at open circuit potential) in the frequency range 100 kHz–50 mHz by applying an alternating current (AC) signal of 10 mV amplitude. For EIS measurements, the results were obtained by applying circular regression to Nyquist experimental plots, using VoltaMaster 4 software. The results give the correlation parameter, which was in the range 0.990 ± 0.008 for all investigated temperatures.

The corrosion inhibition efficiency (*IE*) was calculated from *R_p_* using Equation (2):

(2) $$IE,\%=\left(1-\frac{R_{p}^{0}}{R_{p}}\right)\times 100$$

where $R_{p}^{0}$ and *R_p_* (Ω × cm^2^) are polarization resistance in 1 M HCl, without and with GV-extract, respectively.

Effect of inhibitor concentration. The carbon steel S235 electrodes were immersed in 1 M HCl containing 0 ppm (blank), 50, 100, 200, 300, and 400 ppm G.A.E. from the GV extract.

Temperature effect: Evaluations were conducted on the corrosion process’s thermodynamic parameters and isotherm mechanism at different temperatures (293, 303, 313, and 323 K) in the thermostated electrochemical cell, in the same experimental setup as previously detailed.

## 3. Results and Discussion

### 3.1. Brief GV Extract Chemical Characterization

In a previous work [27], the authors investigated the antioxidant capabilities, chemical analysis, and potential green applications of the ethanol extract derived from *Galium verum* (Yellow Bedstraw), using several techniques to determine antioxidant activity, total polyphenol (Folin–Ciocalteu method), total flavonoids, reverse-phase high-performance liquid chromatography (RP-HPLC), and gas chromatography mass spectrometry (GC-MS) to identify the chemical composition of GV extract.

A short but necessary resume of this previous work [27] is presented below to underline the arguments for choosing GC-extract as a potential corrosion inhibitor.

**Figure 1:**
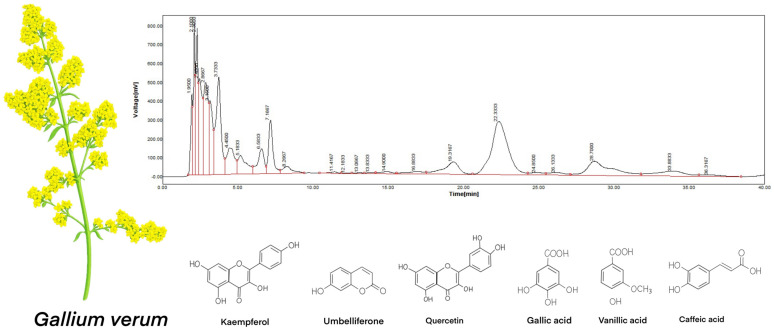
RP-HPLC chromatographic analysis of the GV extract [27].

The primary focus was on the bioactive properties of the ethanol extract, supported by advanced comprehensive chromatographic analysis methods such as RP-HPLC and GC-MS analyses in order to identify critical metabolites responsible for bioactivity in GV extract (Figure 1). Table 1 presents, for RP-HPLC, the identified and dosed polyphenols [27].

Among the components of GV extract identified with GC-MS chromatography, there are alcohols, phenols, carboxylic and fatty acids, esters, aldehydes, and hydrocarbons. In Table 1, for GC-MS, the chemical species present in concentrations over 0.1% are listed.

Substances such as polyphenols were found in abundance, contributing to the extract’s efficacy.

This research explored potential sustainable applications for this extract, ranging from dermatocosmetics to corrosion inhibition. These applications leverage the eco-friendly and biodegradable nature of plant-based products.

### 3.2. Electrochemical Experimental Results at 20 °C (293 K)

#### 3.2.1. OCP Assay

**Figure 2:**
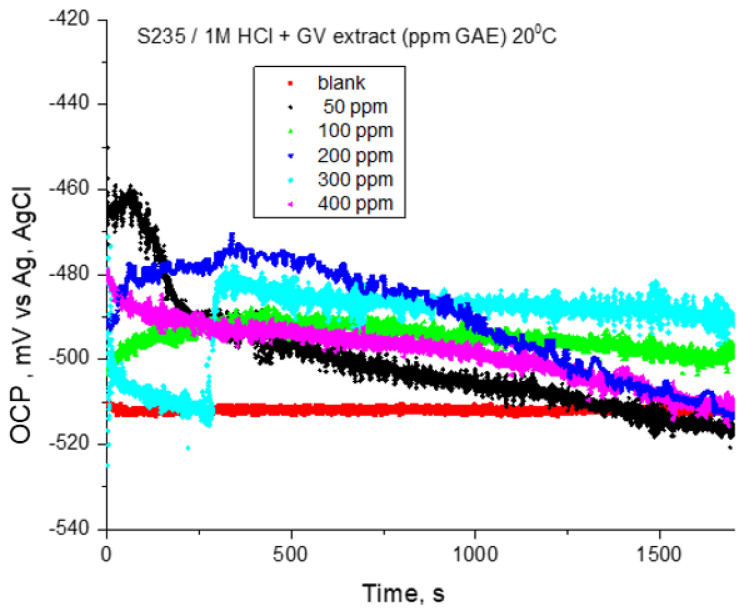
OCP vs. time for carbon steel in 1 M HCl solution with various concentrations of GV extract at 20 °C.

Prior to the potentiodynamic polarization test, the open circuit potential for steel electrodes in 1 M HCl solution in the absence and presence of different concentrations of GV extract was recorded for 30 min. The presence of GV extract slightly shifted the potential towards more positive values. First, after 200 s from electrode immersion, a displacement of OCP towards negative values is observed in all recorded curves (Figure 2). The oxide film’s early dissolving is most likely the cause of the behavior. Additionally, OCP stays rather consistent throughout all inhibitor doses, with the exception of the curves, where a slow rise in OCP may be a sign of a potential film-forming on the electrode surface.

#### 3.2.2. Tafel PP Assay

**Figure 3:**
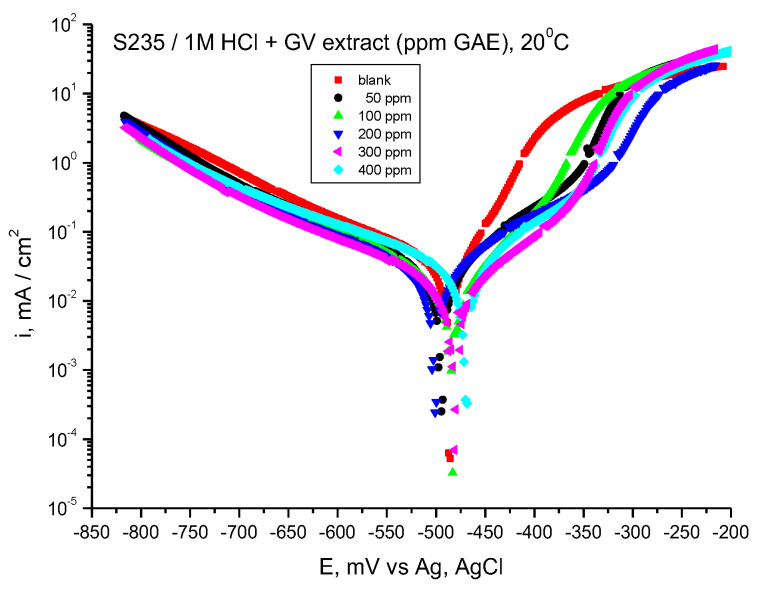
Tafel curves for steel in HCl 1 M with various concentrations of GV extract, 20 °C.

Figure 3 displays the findings from the Tafel measurements.

The corrosion parameters calculated from Tafel plots are presented in Table 2, where *E_corr_* is corrosion potential (V), *i_corr_* is corrosion current density (µA/cm^2^), and *b_a_* and *b_c_* are Tafel anodic and cathodic slopes, respectively (mV/dec).

It is worthy to note that the cathodic branches of the curves are close (the presence of GV extract lowers slightly the cathodic curves), but the influence on the anodic curves is more important, which indicates that GV extract acts mainly on the anodic reaction, steel dissolution.

If the displacement of corrosion potential is lower than 85 mV, the inhibitor is a mixed type. Our results lead to displacement values lower than 85 mV, suggesting that GV is a mixed type of inhibitor, having an influence on both anodic and cathodic reactions.

By examining the data presented in Table 2, it becomes evident that the values for corrosion current density (*i_corr_*) diminish with the incorporation of the GV extract. The most significant efficiency, determined through Equation (1), corresponds to the addition of 500 ppm GV. Furthermore, the anodic (*b_a_*) and cathodic (*b_c_*) Tafel slopes exhibited variations in the presence of the GV extract.

#### 3.2.3. EIS Assay

**Figure 4:**
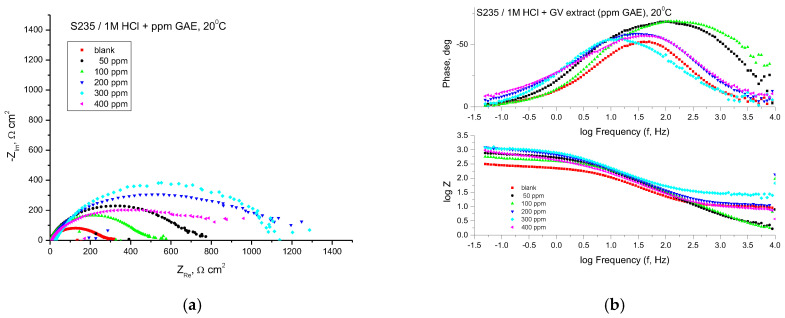
Nyquist (**a**) and Bode (**b**) plots recorded on mild steel in 1 M HCl at various concentrations of GV extract at 20 °C.

The EIS technique was used for studying the corrosion behavior of steel in 1 M HCl solutions with different concentrations of GV extract. Figure 4 shows the Nyquist and Bode plots obtained for mild steel samples in 1 M HCl in the absence and presence of GV extract at 20 °C.

The Nyquist plots consistently exhibited a depressed semicircular pattern, indicating that the corrosion mechanism is predominantly governed by charge transfer phenomena. This distinctive shape can be attributed to the heterogeneity and surface roughness of the analyzed sample. The semicircle diameter is increasing in the presence of GV extract.

The EIS parameters are shown in Table 3, where R_s_ is the solution resistance, *R_p_* is the polarization resistance, *C_dl_* is the double-layer capacitance, and *IE* is the inhibition efficiency calculated using Equation (2).

Table 3 reveals that the polarization resistance exhibits an increase when GV extract is present. Moreover, the most remarkable inhibition efficiency is achieved at a concentration of 500 ppm GAE.

The correlation implies that the polarization resistance (*R_p_*) can be derived by computing the difference between impedance values recorded at high frequencies and those obtained at low frequencies.

(3) Rp=Z Re at low frequency−ZRe (at high frequency)

where *Z_Re_* is the real impedance component (Ω × cm^2^).

A high value of polarization resistance indicates an increased resistance to corrosion. The values of the capacity of the double layer, *C_dl_* (µF/cm^2^), were calculated at the frequency *f_max_*, at which the imaginary component of the impedance is maximum (−*Z_max_*) with the formula:

(4) $$C_{dl}=\frac{1}{2\pi f_{\max}R_{p}}$$

The impedance data presented in Table 3 show a tendency of continuous variation of the *R_p_* values and the inhibition efficiencies (IE, %) depending on the concentration of the GV extract and all indicate an inhibition of the corrosion process in the presence of the GV extract.

With all concentrations of the investigated extract, the Bode representations show that there is only one time constant, which corresponds to the flattened semicircles in the Nyquist representations.

The highest value of inhibition efficiency was obtained in the case of the GV extract with the concentration of 400 ppm G.A.E.

The protective layer’s thickness enhances as the inhibitor concentration rises, owing to the increased electrostatic adsorption of molecules onto the electrode surface. This phenomenon can lead to a notable reduction in the double-layer capacitance (*C_dl_*), aligning with the Helmholtz model, as described by the following equation:

(5) $$C_{dl}=\frac{\varepsilon_{r}\cdot \varepsilon_{0}\cdot A}{d}$$

where *d* is the thickness of the protective layer, *ε_r_* is the relative dielectric constant of the medium, *ε_0_* is the vacuum permittivity, and *A* is the surface area of the electrode. The value of the double-layer capacitance (*C_d_*_l_) decreases when the inhibitor is present, which can be attributed to the successful adsorption of the inhibitor on the surface.

By adding the inhibitor, the impedance modulus increases significantly with the increase of the inhibitor concentration, which denotes an increase in the inhibition efficiency correlated with the thickness of the protective film. This behavior illustrates two different contributions: the first, at high frequencies, can be associated with the formation of the film on the electrode surface, and the second, the charge transfer at the interface. The larger the diameter of the semicircles, the denser the adsorbed monomolecular layer, which leads to an increase in the polarization resistance values and a decrease in the double-layer capacity values.

### 3.3. Electrochemical Experimental Results at 30 °C (303 K)

#### 3.3.1. OCP Assay

**Figure 5:**
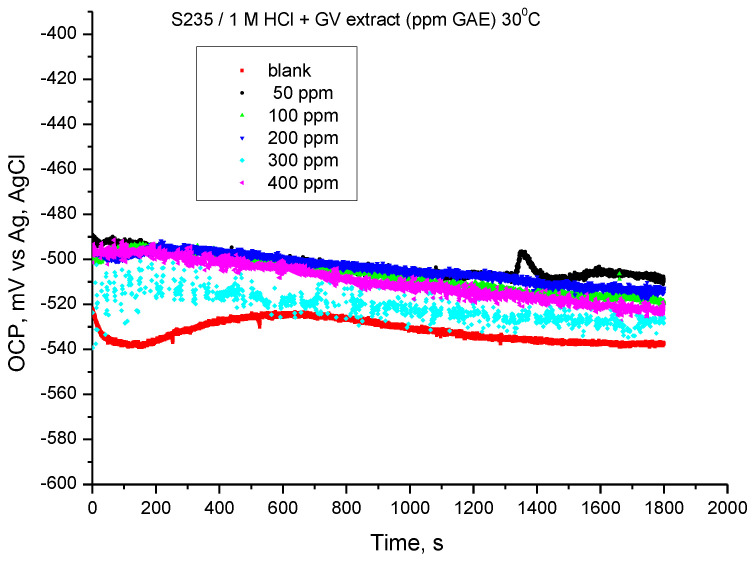
OCP vs. time for carbon steel in 1 M HCl solution with various concentrations of GV extract at 30 °C.

The results of the monitoring of the steel potential in 1 M HCl solution at a temperature of 30 °C are shown in Figure 5. The values of the open circuit potential (OCP) were obtained after 30 min of immersing the electrode in the investigated solutions.

The presence of GV extract influences the OCP value at all concentrations, the tendency being towards more electropositive values. The results suggest that the addition of *Galium verum* extract can achieve corrosion inhibition for S235 carbon steel in 1 M hydrochloric acid solution.

#### 3.3.2. Tafel-PP Assay

**Figure 6:**
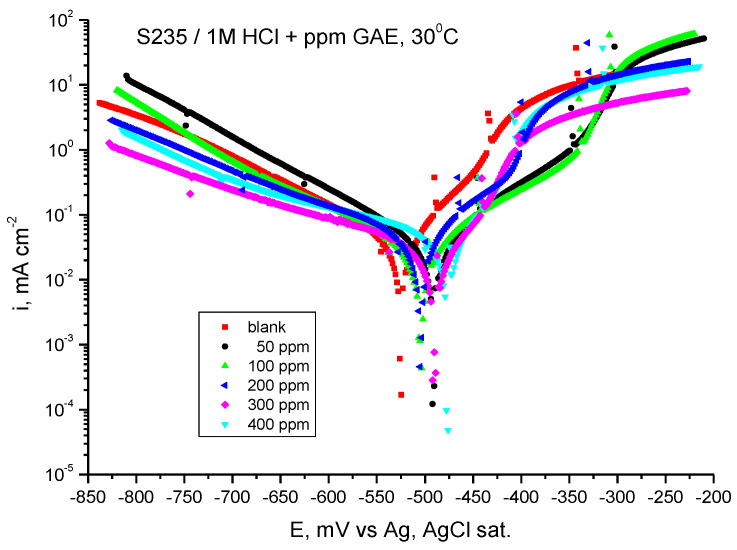
Tafel curves for steel in HCl 1 M with various concentrations of GV extract at 30 °C.

Figure 6 shows the potentiodynamic polarization curves for carbon steel in 1 M HCl solution in the presence of GV extract at 30 °C.

The corrosion potential (*E_corr_*), corrosion current density (*i_corr_*), anodic and cathodic Tafel slopes (*b_a_, b_c_*), corrosion rate, and inhibition efficiency—all of which were derived from the processing of the potentiodynamic polarization curves—are shown in Table 4.

As can be seen in Figure 6, adding GV extract determines a decrease in the corrosion current density at all added extract concentrations. Moreover, in the presence of the studied plant extract, the value of the corrosion potential shifts towards more electropositive values.

As can be seen from Table 4, the anodic and cathodic Tafel slopes (*b_a_* and *b_c_*) vary with the addition of GV extract, being an indication that the inhibition action takes place by blocking the anodic and cathodic active areas on the metal surface. At this temperature, the cathodic slope shows the greatest variance, indicating that the inhibitor is cathodic type.

It is evident from the computed inhibitory efficiency that the extract, with 400 ppm G.A.E., yields the greatest value, 80.56%, at the highest concentration under investigation.

#### 3.3.3. EIS Assay

The corrosion behavior of carbon steel in a 1 M HCl solution containing GV extract was further analyzed using the electrochemical impedance spectroscopy (EIS) technique. This investigation was conducted at a temperature of 30 °C following a 30-min immersion period of the electrode in the solution.

**Figure 7:**
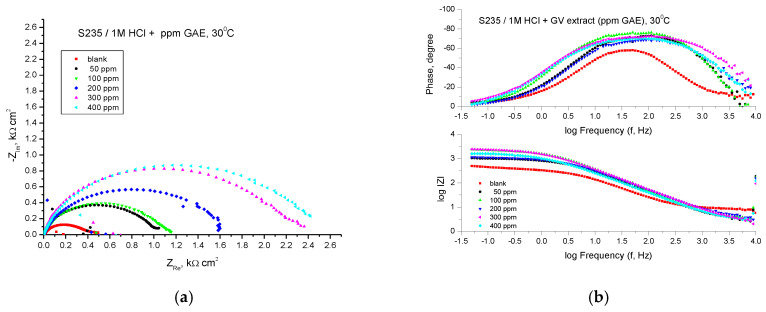
Nyquist (**a**) and Bode (**b**) plots recorded on mild steel in 1 M HCl at various concentrations of GV extract at 30 °C.

Figure 7 shows the results of the EIS experiments in the form of Nyquist and Bode diagrams at the open circuit potential.

Analyzing the shape of the obtained curves, it can be observed that in the case of the addition of the studied plant extract to an acid solution, the obtained curves are also formed in this case by a single capacitive semicircle, suggesting that the corrosion process was controlled by the charge transfer process and at higher temperature. The general shape of the curves is similar for all curves, indicating that the corrosion mechanism does not change in the presence of the inhibitor. The diameter of the semicircles increases with the addition of plant extracts at different concentrations.

The electrochemical parameters obtained from the Nyquist plots are presented in Table 5. The impedance data presented in Table 5 shows a trend of continuous variation of *R_p_* values and inhibition efficiencies (*IE*, %) with increasing concentration of GV extract.

The Bode representations in the presence of all concentrations of the studied GV extract indicate the presence of a single time constant corresponding to the flattened semicircles obtained in the Nyquist representations. The highest value of the inhibition efficiency was also obtained in the case of the extract with the concentration of 400 ppm G.A.E., respectively 86.14%.

### 3.4. Electrochemical Experimental Results at 40 °C (313 K)

#### 3.4.1. OCP Assay

**Figure 8:**
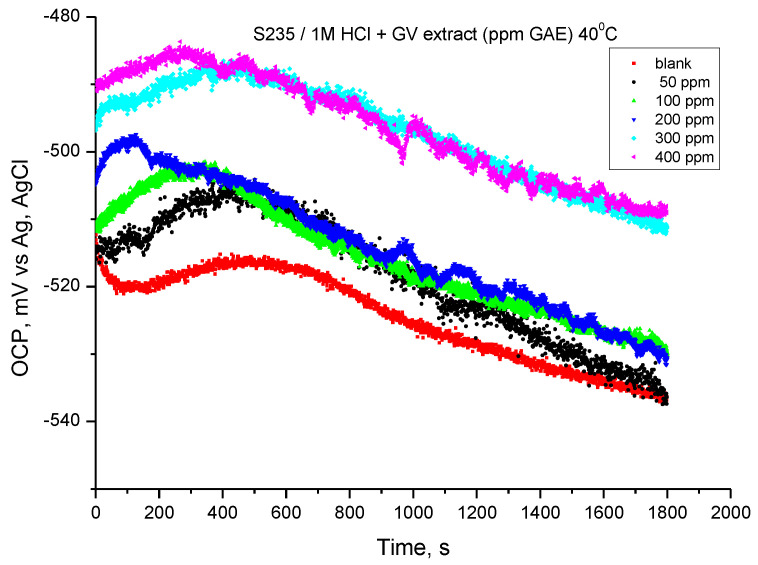
OCP vs. time for carbon steel in 1 M HCl solution with various concentrations of GV extract at 40 °C.

The variation of the open circuit potential of the steel in 1 M HCl solution, recorded at a temperature of 40 °C, is shown in Figure 8. The values resulting from monitoring the open circuit potentials were obtained after 30 min of immersing the electrode in the investigated solutions.

At the temperature of 40 °C, the potential in the presence of GV extract changes their value towards more electropositive values. Initially, the variation in the first 450 s tends towards more electropositive values, then the potential has a shift (similar for all samples) towards more electronegative values. The results suggest that the addition of GV extract can achieve corrosion inhibition for carbon steel in hydrochloric acid solution.

#### 3.4.2. Tafel-PP Assay

**Figure 9:**
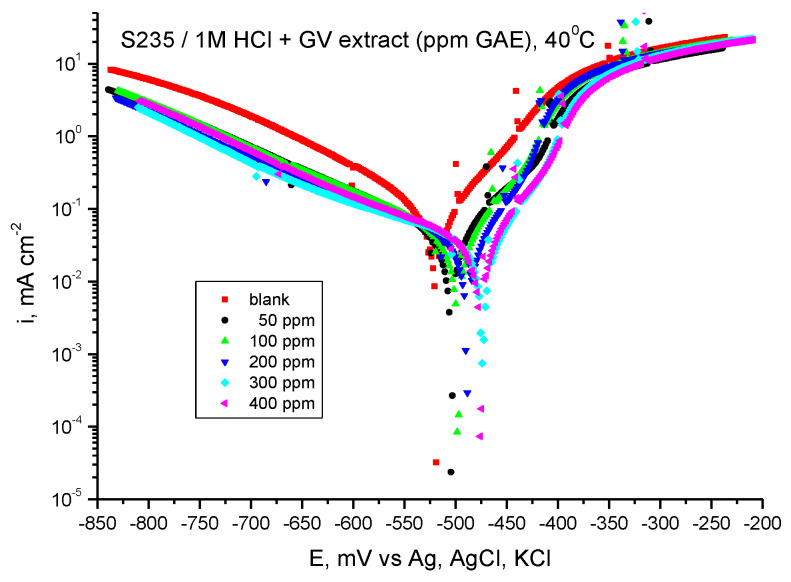
Tafel curves for steel in HCl 1 M with various concentrations of GV extract at 40 °C.

Figure 9 shows the potentiodynamic polarization curves for carbon steel in 1 M HCl solution in the presence of GV extract at 40 °C, and Table 6 shows the results of processing the potentiodynamic polarization curves.

As can be seen from the graphs in Figure 9 and the results presented in Table 6, the presence of *Galium verum* extract leads to a decrease in the ichor corrosion current density at all extract concentrations. Also, in the presence of the studied plant extract, the Ecor corrosion potential value moves towards more electropositive values compared to the value obtained in the absence of the GV extract. The highest values of inhibition efficiency are obtained at the concentration of 400 ppm, followed by those at the concentration of 300 ppm G.A.E.

Therefore, the addition of GV extract affects the speed of the hydrogen release reaction and, implicitly, the corrosion speed of the steel even at a temperature of 40 °C.

It is evident from the computed inhibitory efficiency that the extract with 400 ppm G.A.E. yields the greatest value, 81.35%, at the highest concentration under investigation.

#### 3.4.3. EIS Assay

The behavior of carbon steel against corrosion in 1 M HCl solution in the presence of GV extract was also investigated by the electrochemical impedance spectroscopy method at 40 °C.

**Figure 10:**
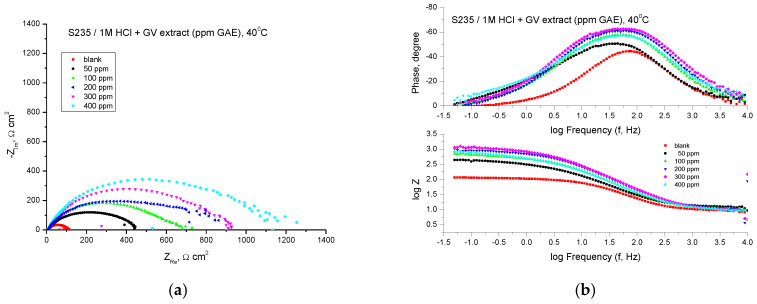
Nyquist (**a**) and Bode (**b**) plots recorded on mild steel in 1 M HCl at various concentrations of GV extract at 40 °C.

Figure 10 shows the results of the EIS experiments in the form of Nyquist and Bode diagrams at the open circuit potential.

From the point of view of the shape of the curves, in the case of the addition of the studied plant extract in acid solution, the obtained curves are formed by a single capacitive semicircle, which suggests that the corrosion process is controlled mainly by the charge transfer process.

The protection against the corrosion of steel in an acid environment given by the inhibitor is evidenced by the increase in the diameter of the semicircles with the addition of the plant extract at different concentrations.

The Bode representations in the presence of all concentrations of the studied extract indicate the presence of a single time constant, which corresponds to the flattened semicircles obtained in the Nyquist representations. The impedance data presented in Table 7 indicate an inhibition of the corrosion process in the presence of GV extract.

The highest value of the inhibition efficiency was obtained in the case of the extract with the concentration of 400 ppm of 91.82%, followed by that of the extract with the concentration of 400 ppm GAE.

### 3.5. Electrochemical Experimental Results at 50 °C (323 K)

#### 3.5.1. OCP Assay

**Figure 11:**
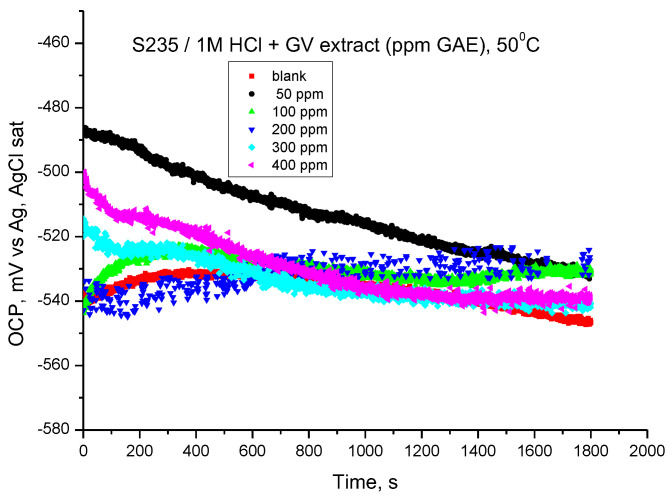
OCP vs. time for carbon steel in 1 M HCl solution with various concentrations of GV extract at 50 °C.

The values of the open circuit potentials were obtained after 30 min in which the electrode was immersed in the investigated solutions, and the values of the Ecor corrosion potentials were determined by extrapolating the Tafel lines drawn in the potentiodynamic regime. The GV extract influences the OCP value at all concentrations (Figure 11); in most cases, the tendency is towards more electropositive values. The results obtained highlight the achievement of corrosion inhibition by the addition of GV extract in HCl medium.

#### 3.5.2. Tafel-PP Assay

**Figure 12:**
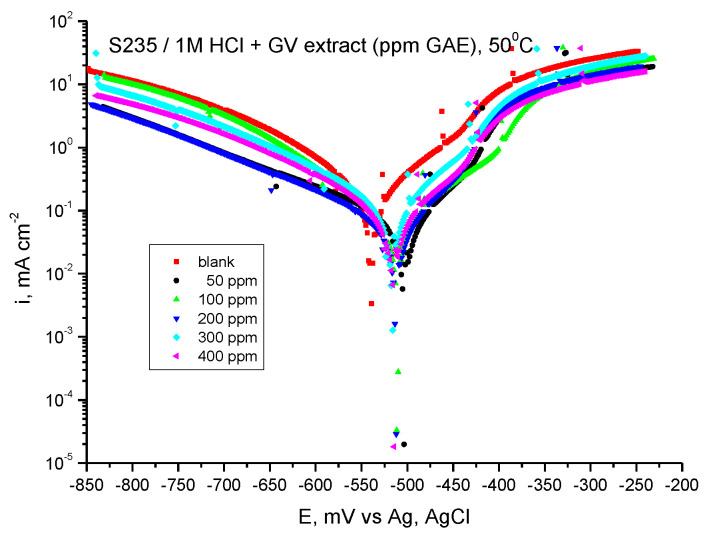
Tafel curves for steel in HCl 1 M with various concentrations of GV extract at 50 °C.

Tafel-potentiodynamic polarization curves for carbon steel in 1 M HCl solution in the absence and presence of GV extract at 50 °C are shown in Figure 12.

The electrochemical corrosion parameters resulting from the polarization curves are presented in Table 8.

In the anodic and cathodic Tafel slopes *b_a_* and *b_c_*, a small variation is observed with the addition of GV extract, thus indicating that the inhibition action takes place by blocking both the anodic and cathodic active areas on the metal surface at about the same proportion.

The highest inhibition efficiency, 79.20%, is also obtained at the maximum studied concentration, 400 ppm GAE.

#### 3.5.3. EIS Assay

The corrosion behavior of carbon steel in 1 M HCl solution in the presence of GV extract was also investigated by the electrochemical impedance spectroscopy method at 50 °C after 30 min of immersion of the electrode in the solution and after the potentiodynamic polarization assay.

**Figure 13:**
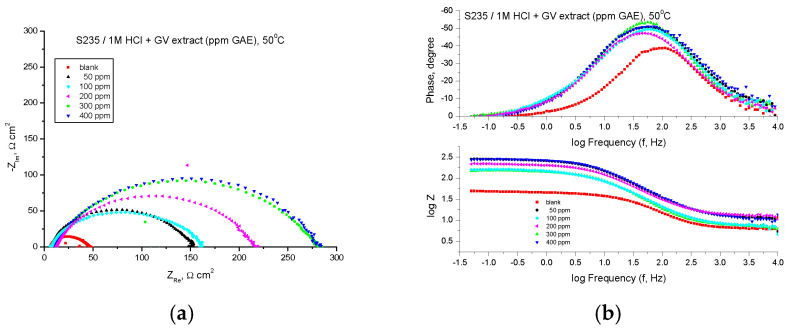
Nyquist (**a**) and Bode (**b**) plots recorded on mild steel in 1 M HCl at various concentrations of GV extract at 50 °C.

The shape of the curves is similar for all determinations (Figure 13), indicating that there is almost no change in the corrosion mechanism due to the action of the inhibitor. The diameter of the semicircles increases with the addition of the plant extract at different concentrations, which proves that the presence of the plant extract protects the steel against corrosion in an acidic environment.

The impedance data presented in Table 9 shows a trend of continuous variation of *R_p_* values and inhibition efficiencies *(IE,* %) depending on the concentration of GV extract, all of which indicate an inhibition of the corrosion process in the presence of GV extract.

The Bode representations in the presence of all concentrations of the studied extract indicate the presence of a single time constant, corresponding to the flattened semicircles obtained in the Nyquist representations. The highest value of the inhibition efficiency was obtained in the case of the extract with the concentration of 400 ppm GAE, 86.40%, the next value being obtained for the extract with the concentration of 300 ppm, 86.36%.

**Figure 14:**
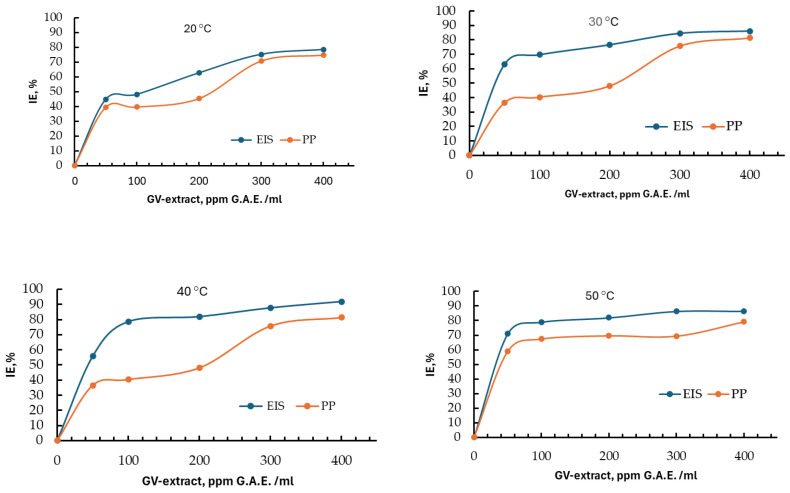
Evolution of inhibition efficiency with GV extract concentration, at different temperatures, obtained with Tafel (PP) and EIS methods.

The influence of concentrations on the efficiency of the inhibitor for the different temperatures studied with the two Tafel (PP) and EIS methods is presented in Figure 14 and indicates at the beginning an increase in efficiency with increasing concentration. It is observed that for the last two values of the GV extract concentration of 300 and 400 ppm GAE, respectively, the growth trend flattens out. This is a logarithmic behavior that tends towards a limit value, which would indicate the concentration of 400 ppm GAE as sufficient for corrosion inhibition of carbon steel in 1 M HCl.

It can also be observed that even for the smallest amount of GV extract inhibitor, the inhibitor efficiency increases remarkably to values over 60%, which confirms that it is an adsorption inhibitor with double anodic and cathodic action.

As can be seen from Figure 14, at a temperature of 50 °C, the inhibitory effect stabilizes much faster than at lower temperatures, which means that the corrosion protection provided by GV extract can be extended to 50 °C, even if the inhibition efficiency is somewhat lower than at 40 °C.

**Figure 15:**
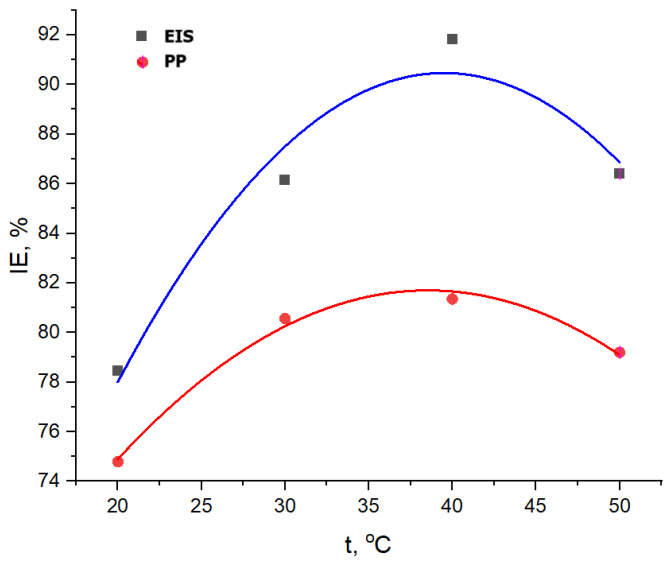
Evolution of inhibition efficiency of GV extract with temperature, obtained with Tafel (PP) and EIS methods.

The effect of temperature on inhibitory efficiency is presented in Figure 15, and it is observed that there is a parabolic dependence, with a maximum point corresponding to the concentration of 400 ppm G.A.E. For comparison, the highest values of *IE*, %, from both PP and EIS methods were chosen for every temperature.

**Figure 16:**
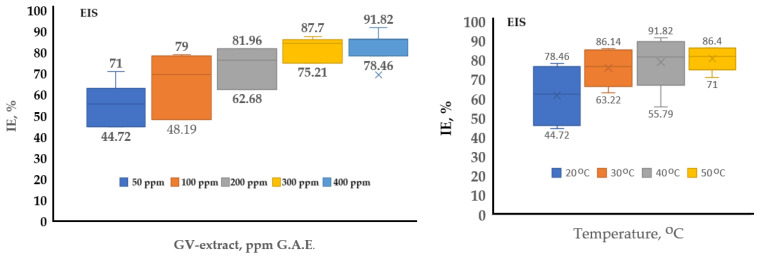
Box charts for comparative characterization of GV extract at different concentrations and temperatures using EIS electrochemical methods of investigation.

In Figure 16, two box-charts have been completed to conclude the inhibition efficiency of GV extract for the studied system, using the EIS electrochemical method of investigations.

The EIS method shows bigger values of *IE* than PP. The higher value of EIS describes better the inhibitor efficiency, because EIS is closely related not only to electrochemical potentials but also to the electrochemical double layer and the corrosion products. Table 10 presents relevant statistical data, including mean values, standard deviation (SD), and coefficient of variation (COV), providing an overview of the variation and trend of the analyzed data. From the COV results, it is observed that the variability of the measurements tends to decrease as the concentration of GV extract increases from approximately 19.07% for the 50 ppm concentration to approximately 6.42% for the 400 ppm concentration, the effect of the extract becoming stronger at higher concentrations.

From Figure 15, the optimum temperature for using GV extract is 40 °C, but taking into consideration Figure 16 and the COV value of 17.7218, which is bigger than the 50 °C, value of 78,815, it could be concluded that the temperature of 50 °C is better, which means that the GV extract corrosion inhibitor could be at its best result even at 50 °C. Over this temperature, the inhibition efficiency becomes lower due to the intrinsic corrosion mechanism, presented below.

This behavior can be explained by the stability of flavonoids at temperature. The effect of temperature on the stability of flavonoids is a topic of interest in scientific research [55,59,60]. Studies suggest that temperature has a significant effect on the stability of flavonoids and on their biological activity. The structure of flavonoids is responsible for the action of temperature on them. For example, glycosylated flavonoids are more resistant to heat treatment compared to aglycones [55]. The evaluation of oligomeric flavonoids’ thermal properties and the correlation between the thermal stability and antioxidant and antimicrobial activity of poly(flavonoids) is a scientific novelty and is an extension of current research on polymeric forms of flavonoids obtained in enzymatic polymerization reactions [55]. Increasing temperature affects the stability of polyphenols.

This study leads to the conclusion that increasing temperature can accelerate the oxidation of polyphenols, leading to the loss of antioxidant activity responsible for inhibiting corrosion. Thus, antioxidant activity does not only refer to biochemical and phytotherapeutic evaluation but becomes a measure of the capacity of a group of compounds (polyphenols) to act as corrosion inhibitors. The stronger the antioxidant activity of a plant extract, the higher the polyphenol content, and the higher the inhibition effect on metal corrosion.

In Table 11 is presented comparatively the inhibition efficiency of a few green inhibitors in HCl 1 M. It can be said that the GV extract is very good; the efficiency is over 90%, comparable with other reported data.

### 3.6. GV Extract—A New Corrosion Eco-Inhibitor

A notable aspect of this work is the focus on *Galium verum*, a plant species previously underexplored from this perspective, or corrosion inhibitor. This study bridges gaps in knowledge, opening avenues for further research on its multifaceted applications.

Comprehensive chromatographic analyses (HPLC, GC-MS) identified critical metabolites responsible for bioactivity [27].

Substances such as kaempferol, umbelliferone, quercetin, gallic acid, vanillic acid, and caffeic acid were found in abundance, contributing to the extract’s efficacy.

This study differs from previous works by using a plant that has not been previously investigated as a corrosion inhibitor. In addition, a detailed characterization of the active substances responsible for the inhibition, comparing their efficiency with that of other natural and synthetic inhibitors. The results obtained are comparable to those reported in the literature, but our contribution consists in highlighting the potential of a completely new and sustainable source.

In Table 12 there is a comparison that highlights that plant-based inhibitors, and so is GV extract, are a promising alternative for sustainable applications, although classical inhibitors remain preferred in stringent industrial environments.

The GV extract has all the advantages of green inhibitors, extracted from plants, but it also has some specific advantages: GC is a spontaneous plant, widespread in Europe, Asia, and North Africa, with no environmental impact. Plant culture could be used both for phytotherapeutic and industrial purposes.

As a corrosion inhibitor in acid media, the efficiency is very good, with a maximum value of 91.82%, close to classical ones.

Results of analytical investigations on ethanolic GV extract proved it to be a polyphenol-based green inhibitor [27]. In comparison with other chemical categories of substances discovered in plant extracts, polyphenols from GV extract act as a mixed inhibitor by forming protective films on metal surfaces. The main known categories of phytochemicals responsible for corrosion inhibition are alkaloids [64], phenolic compounds [65], tannins [66], and triterpenes [64]. The complex chemical compositions of these plant extracts make it very difficult to attribute the inhibitory action to a particular component or group of components. Thus, phenolic compounds generated more interest because of their high redox potential, which allows them to act as reducing agents, hydrogen donors, and singlet oxygen inhibitors, and also because of their metal chelating potential [1]. In Table 13 a comparison between different chemical categories of plant inhibitors is presented.

Detailed explanation of polyphenol action as corrosion inhibitors is given below (Section 3.7).

Derived from renewable natural resources, the GV extract could be a part of a circular economy and reduce the utilization of classical, non-renewable, synthetic inhibitors. Because the plant is readily available, it will involve low production costs. Being already part of pharmacopeia, not only in Eastern Europe but also in other regions, GV provides fewer risks to human health or the ecosystem.

The research also explored potential sustainable applications for this extract, leveraging the eco-friendly and biodegradable nature of plant-based products.

The findings pave the way for future studies on enhancing extraction and/or investigation techniques and broadening applications to include not only therapeutic but also industrial fields.

These advantages make GV extract, a plant-based inhibitor, a promising solution for industries looking to adopt greener and more sustainable practices.

### 3.7. Consideration on Inhibitor Corrosion Mechanism of GV Extract

From the electrochemical experimental results, *Galium verum* extract was shown to be a mixed type of inhibitor that acts on both the anodic metal dissolution reaction and the cathodic hydrogen evolution reaction.

At carbon steel corrosion in an acidic medium, a sequence of steps could be identified in the corrosion mechanism [61]. Thus, the next electrode processes could be mentioned:

- The anodic carbon steel dissolution reactions:

(6) $$Fe+H_{2}O\to Fe{\left(OH\right)}_{ads}+H^{+}+e^{-}$$

(7) $$Fe{\left(OH\right)}_{ads}\to Fe{\left(OH\right)}^{+}+e^{-}$$

(8) $$Fe{\left(OH\right)}^{+}+H^{+}\to {Fe}^{2+}+H_{2}O$$

- The cathodic hydrogen evolution reactions:

(9) $$Fe+H^{+}\to {\left(FeH^{+}\right)}_{ads}$$

(10) $${\left(FeH^{+}\right)}_{ads}+e^{-}\to {\left(FeH\right)}_{ads}$$

(11) $${\left(FeH\right)}_{ads}+H^{+}+e^{-}\to Fe+H_{2}↑$$

On the steel surface a film is formed by the adsorption of GV extract, by the interaction of iron ions and plant extract compounds.

Absorption inhibitors are substances that can selectively adsorb either the anodic zones or the entire metal surface, forming passivation films that slow down the dissolution of metal or stop the diffusion of the depolarizer in the cathodic areas.

Adsorption inhibitors are polar molecules that adsorb on the metal surface, inhibiting both electrode processes but especially the cathodic process of hydrogen discharge. These inhibitors are particularly effective in very small quantities (10^−5^ ÷ 10^−3^ mol/L) because they act in almost monomolecular layers. The greatest effectiveness of adsorption inhibitors is in acidic media.

**Figure 17:**
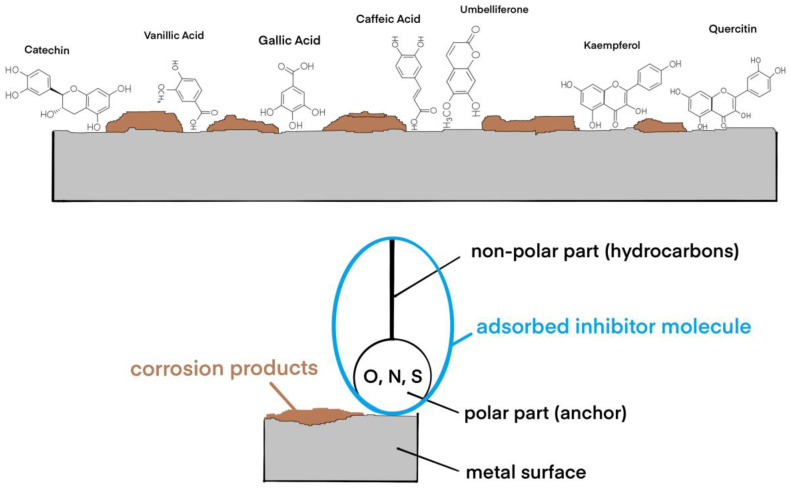
Schematic representation of the adsorption of corrosion inhibitors competing with corrosion products on the metal surface.

Adsorption of inhibitors is a particular case, electrosorption occurring through a substitution reaction of solvent molecules oriented towards the metal surface, and it is also influenced by the electrical variable of the interface, i.e., the electrode potential. Adsorption of organic molecules influences the surface tension and the differential capacitance of the electrochemical double layer, their maximum adsorption occurring near zero charge. In practice, adsorption inhibitors are surface-active substances or surfactants (Figure 17).

The adsorption mechanism is dependent on the polarizability of the molecules. The asymmetric chemical structure, formed by two parts, one polar—acting as an anchor—and one non-polar, oriented towards the corrosive environment, is formed by the hydrocarbon residue and forms the actual adsorption layer, which isolates the metal and prevents the corrosion reaction. Among the most effective anchor functional groups are -CH_2_-OH, -COOH, -CHO, and -COOR, molecules that are found in abundance in the GV extract. In a previous study [27], a chemical characterization of *Galium verum* extracts using analytical techniques identified and measured the presence of polyphenols: gallic acid, catechin, vanillic acid, caffeic acid, kaempferol, quercetin, and umbelliferone, and other compounds including alcohols, phenols, fatty acids, hydrocarbons, esters, and other aromatic compounds.

Size, spatial orientation, shape, and electrical charge play an important role in the inhibition mechanism. The larger the volume of a molecule (aromatic compounds, for example), the greater the inhibition capacity. The nature of the metal is also important. Metals in the Fe group, having free “d” electronic levels, tend to desorb organic molecules with “*π*” electrons, i.e., polar molecules, containing O, N, and S.

Also, the greater the number of polar groups, as in the case of polyphenols, the more intense the corrosion inhibition.

Taking into consideration the above arguments, among the substances present in plant extract to be corrosion inhibitors, polyphenols could be considered the main actors in limiting the corrosion rate of metals.

## 4. Conclusions

Due to the variety of existing plants and their constituent substances, in order to replace the dangerous toxic substances that have proven their effectiveness in protecting metals against corrosion, studies are being carried out to test plant extracts against corrosion. Most extracts are prepared from leaves, but they have also been reported for other parts of plants. Acidic environments are the most tested corrosive environments, being encountered in various industrial processes, and carbon steel is one of the most widely used industrial materials; therefore, the protection of carbon steel is the most researched. The use of natural corrosion inhibitors, obtained from plants, brings significant environmental benefits. These biodegradable compounds reduce pollution and risks associated with synthetic chemicals, which can be toxic and difficult to break down. Furthermore, extracting inhibitors from plant sources promotes a sustainable process, avoiding the contamination of soil and water with hazardous substances. This approach helps protect ecosystems and reduce the negative impact on human health and nature. In essence, choosing nature is an important step towards a greener future.

In this work, the inhibitory effect of the extract of *Galium verum* (GV) was evaluated as a corrosion inhibitor for carbon steel in 1 M HCl solutions. Studies were carried out in the temperature range 20–50 °C.

The determinations of the potential in the open circuit made at all the studied temperatures showed the displacement of the potential towards more positive values in the solutions with the addition of GV extract compared to the results obtained in the control solution of 1 M HCl.

The results of the Tafel potentiodynamic polarization studies showed that the presence of the GV extract changed the corrosion potential towards more positive values, and the variation of the cathodic slopes was more pronounced than the anodic slopes, suggesting that the GV extract is a mixed inhibitor, predominantly cathodic. Electrochemical impedance spectroscopy studies showed an increase in polarization resistance in the presence of GV extract compared to the results obtained in its absence.

Green inhibitors have been reported to have an inhibition efficiency around 60–95%, which means that *Galium verum* is a very good one. The corrosion inhibitory efficiency of the *Galium verum* ethanolic extract had a maximum value of 91.82%, for a concentration of 400 ppm polyphenol content, demonstrating the inhibitory potential of this green product comparable with synthetic inhibitors for steel in acid media. Obtained values of the inhibition efficiencies for mild steel in 1 M HCl were between 19.92% at the addition of 50 ppm GAE at 30 °C and 91.82% at 400 ppm GAE at the temperature of 40 °C. Temperatures over 40 °C decrease the inhibition efficiency of GV extract due to less stability of polyphenols, the chemical species responsible for the inhibition effect. Polyphenols and other polar molecules contained in GV extract are adsorption corrosion inhibitors, acting after a mixed mechanism.

The presence of the GV extract led to the reduction of the corrosion rate, the results being comparable to the values obtained for other plant extracts, over 90%, therefore promising for the use of the GV extract as a green inhibitor of steel corrosion in acid.

GV extract proved to be a new corrosion inhibitor with specific advantages: spontaneous flora, widespread on the globe, sustainability, cost-effectiveness, high inhibitor efficiency, and reduced health risks.

Electrochemical, analytical, and statistical techniques from this study establish the mechanism of corrosion and the chemical species involved but are not exhaustive; further investigations will be continued, and more powerful methods should be used to provide additional information to analyze in detail the interaction of the inhibitor with the metal surface.

## Figures and Tables

**Table 1:** Main chemical components identified in GV extract by analytical techniques [27].

Analytical Method	Chemical Compounds
RP-HPLC	kaempferol, umbelliferone gallic acid quercetin catechin vanillic acid caffeic acid,
GC-MS (over 0.1%)	Palmitic acid Linoleic acid 2-Methyl-benzene Linolenic acid Methanol Butyric acid. octyl ester 3-O-Methyl-d-glucose 1,3,6-Trideoxy-3,6-epithio-D-Fructose Benzyl benzoate Phytol 2,3,4-Trimethyloxetane 5-Hydroxy-methyl-furfural Stearic acid

**Table 2:** Tafel-derived kinetic parameters for steel in 1 M HCl at various concentrations of GV extract at 20 °C.

HCl 1 M + GV Extract (ppm G.A.E), at 20 °C	*E_corr_*, (mV/SCE)	*i_corr_*, (µA/cm^2^)	*R_p_*, (Ω × cm^2^)	*b_a_*, (mV/dec)	−*b_c_*, (mV/dec)	*IE*, %
0	−492	28.278	543.42	53.0	132.4	-
50	−507	17.122	887.72	80.1	105.3	39.45
100	−500	17.064	809.15	70.8	109.7	39.66
200	−490	15.447	996.38	86.6	104.6	45.37
300	−489	8.270	1270	74.5	87.9	70.75
400	−480	7.127	1500	58.7	78.5	74.79

**Table 3:** EIS parameters for steel corrosion in 1 M HCl solution at various concentrations of GV extract at 20 °C.

HCl 1 M + GV Extract (ppm G.A.E.), at 20 °C	*R_s_*, (Ω × cm^2^)	*R_p_*, (Ω × cm^2^)	*C_dl_*, (µF/cm^2^)	*IE*, %
blank	8.96	239.5	132.8	-
50	4.11	475.9	125.4	44.72
100	4.65	462.3	117.1	48.19
200	6.22	641.8	116.5	62.68
300	8.52	966.2	107.2	75.21
400	25.11	1112	101.8	78.46

**Table 4:** Tafel-derived kinetic parameters for steel in 1 M HCl at various concentrations of GV extract at 30 °C.

HCl 1 M + GV Extract (ppm G.A.E.), at 30 °C	*E_corr_*, (mV)	*i_corr_*, (µA/cm^2^)	*R_p_*, (Ω × cm^2^)	*b_a_*, (mV)	−*b_c_*, (mV)	*v_corr_*, (µm/Y)	*IE*, %
blank	−530	52.1491	471.99	90	220	403.0	-
50	−496	33.0671	486.57	79	122	366.1	36.59
100	−510	31.0798	510.61	61	126	238.4	40.40
200	−496	27.0590	595.33	72	93	179.8	48.11
300	−509	12.6644	806.01	80	149	166.5	75.72
400	−529	9.7264	749.23	70	84	126.9	80.56

**Table 5:** EIS parameters for steel corrosion in 1 M HCl solution at various concentrations of GV extract at 30 °C.

1 M HCl + GV Extract (ppm G.A.E.), at 30 °C	*R_s_*, (Ω × cm^2^)	*R_p_*, (Ω × cm^2^)	*C_dl_*, (µF/cm^2^)	*IE*, %
blank	6.64	371.8	119.8	-
50	0.25	1011.0	86.89	63.22
100	0.87	1229.0	64.08	69.74
200	2.48	1591.0	62.92	76.63
300	0.98	2411.0	58.30	84.57
400	4.65	2683.0	53.20	86.14

**Table 6:** Tafel-derived kinetic parameters for steel in 1 M HCl at various concentrations of GV extract at 40 °C.

HCl 1 M + GV Extract (ppm G.A.E.), at 40 °C	*E_corr_*, (mV)	*i_corr_*, (µA/cm^2^)	*R_p_*, (Ω × cm^2^)	*b_a_*, (mV)	−*b_c_*, (mV)	*v_corr_*, (µm/Y)	*IE*, %
blank	−530	52.1491	471.99	90	220	403.0	-
50	−496	33.0671	486.57	79	122	366.1	36.59
100	−510	31.0798	510.61	61	126	238.4	40.40
200	−496	27.0590	595.33	72	93	179.8	48.11
300	−509	12.6644	806.01	80	149	166.5	75.72
400	−529	9.7264	749.23	70	84	126.9	81.35

**Table 7:** EIS parameters for steel corrosion in 1 M HCl solution at various concentrations of GV extract at 40 °C.

1 M HCl + GV Extract (ppm G.A.E.), at 40 °C	*R_s_*, (Ω × cm^2^)	*R_p_*, (Ω × cm^2^)	*C_dl_*, (µF/cm^2^)	*IE*, %
blank	8.949	94.28	106.6	-
50	8.119	213.24	211.9	55.79
100	8.329	440.20	180.7	78.58
200	9.272	520.00	76.51	81.87
300	9.397	766.40	58.14	87.70
400	8.160	1153.00	68.96	91.82

**Table 8:** Tafel-derived kinetic parameters for steel in 1 M HCl at various concentrations of GV extract at 50 °C.

HCl 1 M + GV Extract (ppm G.A.E.) at 50 °C	*E_corr_*, (mV)	*i_corr_*, (µA/cm^2^)	*R_p_*, (Ω × cm^2^)	*b_a_*, (mV)	−*b_c_*, (mV)	*v_corr_*, (µm/Y)	*IE*, %
blank	−538.51	113.567	103.41	75.6	77.5	1071	-
50	−501.19	46.519	176.13	69.3	131.4	543.5	59.04
100	−513.02	36.921	272.06	72.8	76.3	371.0	67.49
200	−513.67	34.473	333.48	66.7	94.6	290.6	69.65
300	−516.91	34.751	378.08	40.6	53.9	286.4	69.40
400	−516.12	23.620	303.70	45.2	48.1	245.1	79.20

**Table 9:** EIS parameters for steel corrosion in 1 M HCl solution at various concentrations of GV extract at 50 °C.

HCl 1 M + Extract (ppm G.A.E.) at 50 °C	*R_s_*, (Ω × cm^2^)	*R_p_*, (Ω × cm^2^)	*C_dl_*, (µF/cm^2^)	*EI*, %
blank	6.155	36.78	154.0	-
50	10.08	141.1	82.61	73.93
100	6.056	155.6	143.1	76.36
200	11.94	203.9	98.31	81.96
300	6.588	269.7	126.3	86.36
400	10.27	270.5	74.10	86.40

**Table 10:** Statistical data for inhibition efficiency at different temperatures for the studied concentrations of GV extract inhibitor, obtained by the EIS method.

EIS							
GV Extract, ppm G.A.E.	Mean	SD	COV	Temperature, °C	Mean	SD	COV
50	58.6825	11.18969	19.06819	20	61.8520	15.2897	24.7199
100	68.8775	14.43745	20.96106	30	76.0600	9.7360	12.8004
200	75.785	9.085022	11.98789	40	79.1520	14.0272	17.7218
300	83.46	5.647483	6.766694	50	80.9440	6.3796	7.8815
400	85.705	5.494103	6.410481				

**Table 11:** Comparative inhibition efficiency of some newly reported plant extracts.

Plant Extract	Inhibition Efficiency, %	[Ref.]
*Galium verum*	91.82	This study
*Urtica dioica*	>95	[53]
*Falcaria vulgaris*	91.3	[54]
*Artemisia Stems*	90	[58]
Marjoram	92	[57]
*Ambrosia trifida*	97.5	[61]
*Convolvulus microphyllus*	92.47%	[62]
*Artemisia herba-alba*	96.17%	[63]

**Table 12:** Comparison of GV extract vs. other plant inhibitors and classical inhibitors.

Corrosion Inhibitor	GV Plant Extract	Plant Extracts	Classical
Origin	Ethanolic extract of aerial parts of *Galium verum*	Hydro and/or alcoholic extracts from different parts of plants (leaves, flowers, seeds, bark)	Synthetic compounds, such as phosphates, chromates, or hydrazine
Efficiency in acid media	91.82%	≥80%	90 ÷ 100%
Advantages	Spontaneous flora Widespread on the globe Sustainability Cost-effectiveness High inhibitor efficiency Reduced Health Risks Phytotherapeutically use	Biodegradable, Environmentally friendly, Less toxic	Stability over time Constant efficiency in various conditions
Disadvantages	Lower chemical stability and lower corrosion efficiency at temperatures over 50 °C	Performance depending on chemical composition and environmental conditions	High toxicity, Negative impact on the environment

**Table 13:** Comparison of corrosion behavior of different types of chemical compounds from plant extracts.

Chemical Category	Media	Corrosion Efficiency	Mechanism	[Ref.]
Alkaloids	acid	≤85%	Interaction with metal surface	[64]
Polyphenols	acid neutral	≥85% ≥80%	Protective films on metal surface	[65]
Tannins	alkaline	≤90%	Forms complexes with metal ions on the metal surface	[66]
Terpenoid	Acid	70 ÷ 80%	Adsorption on metal surface	[64]

## Data Availability

Data is contained within the article.
